# Case of Occlusion of the Vagina in a Pregnant Woman

**Published:** 1845-04

**Authors:** A. Davizac

**Affiliations:** New Orleans


*Case of Occlusion of the Vagina in a pregnant woman.* By A. Davizac, M. D., *of New Orleans*.—(This case was reported to the Medico-Chirurgical Society of Louisiana, in October, 1843.—Ed'rs.)

A few days since I visited, in consultation with Dr. B. of this city, a woman under his care, aged about twenty-six years, of robust stature, somewhat plethoric habit, and muscular appearance, at the time laboring under strong expulsive pains of childbirth. I proceeded to the examination *per vaginam*, and to my astonishment found a little above the mouth of the urethra, a complete obstruction to the passage of the finger. Upon a minute examination, the vagina was found apparently completely closed by a strong, dense, striated membrane—the striæ radiating from the centre, and giving the sensation of tense cords. Finding that delivery was impracticable, Dr. B. and myself concluded to administer an anodyne in order to lull her pains; and advised her husband to call in Dr. Luzenberg the following morning. She slept well and remained quiet during the remainder of the night.

Dr Luzenberg met us early the next morning, and satisfied himself of the condition of the parts above described; with the addition of an opening not larger than would admit the head of an ordinary probe, which could only be observed when the membrane was protruded by the head of the child during the expulsive pain. At noon of the same day, he performed an operation by making a crucial incision the whole extent of the obstructing membrane. It proved to be very thick and tough, resisting the instrument like tendon. Immediately after the operation the head of the fœtus could be distinctly defined; the occiput presenting itself to the left of the pubis. The labour then appeared to advance tolerably well, and we thought would be soon terminated. At night the head had descended so as to press considerably on the perineum; but still the soft parts continued rigid and unyielding, and the bones of the head preserved their spherical form.

We then attempted to deliver with the forceps, and with difficulty introduced one blade, but we found the head so firmly impacted that we did not deem it prudent to proceed any further, and desisted.

On the following morning, at the suggestion of Dr. L. we determined to perforate the head, and attempt to bring it away with the hook. Accordingly we placed the patient on a table for the purpose of operating with more facility—and having passed the perforator into the fontanel, and broken up the brain, the bones of the cranium collapsed, and then by the use of considerable force with the hook, we were enabled to extract the head. After this, the shoulders offered great resistance, but we finally succeeded in bringing away an uncommonly large male infant. It had been sometime dead, as was indicated by the livid spots and phlyctanæ on the neck. The placenta followed immediately afterwards. The uterus contracted well and quickly; and the patient complained of nothing now but exhaustion. She is at this time convalescent.

Upon inquiring minutely into the previous history of this case, it was ascertained that a few years since, she was married the first time —that she had been a stout and healthy girl—that about a year after marriage she was confined in childbed, and had such a difficult labour that the fœtus had to be dismembered before delivery could be effected; and that she was very much bruised and lacerated during the operation. Very soon after her accouchement, and before she had any intercourse with her husband, he died. She slowly recovered, and had no sexual intercourse until her marriage to her present husband, which took place about two years afterwards. She menstruated regularly during the time. The husband says there seemed to be considerable difficulty in the first copulation, but that since then, although he always perceived an obstacle, it seemed to be elastic, and yielding to a considerable degree. Her last accouchement took place about 16 months after marriage.

Upon reviewing the history of the case, it would appear that owing to the laceration caused by the first instrumental delivery, there was an adhesion of the walls of the vagina, or the formation of an adventitious membrane which closed it up with the exception of a very small perforation, which could only be discovered when it was distended, but was sufficient to admit the discharge of the menses, and the ingress of the *semen masculinum.* The obstructing membrane was sufficiently elastic to admit the male organ into the vagina to a satisfactory extent. The woman has a remarkably narrow pelvis, and it is to be apprehended can never be delivered without instruments.

The most interesting features of this case relate to the formation, and the extremely small perforation of this adventitious membrane, (being not larger than the end of a probe ;) yet it seems to have been sufficient to allow egress to the menstrual flux, and consequently must have admitted some portion of seminal fluid ; yet the latter must have been greatly obstructed in *its natural jet*, and could scarcely have penetrated as far as the *os uteri*. Such however are the facts in the case, and they are submitted to the society.—*Ibid.*

